# Candidate odorant binding proteins and chemosensory proteins in the larval chemosensory tissues of two closely related noctuidae moths, *Helicoverpa armigera* and *H*. *assulta*

**DOI:** 10.1371/journal.pone.0179243

**Published:** 2017-06-08

**Authors:** Hetan Chang, Dong Ai, Jin Zhang, Shuanglin Dong, Yang Liu, Guirong Wang

**Affiliations:** 1 College of Plant Protection, Nanjing Agricultural University/Key Laboratory of Integrated Management of Crop Diseases and Pests (Nanjing Agricultural University), Ministry of Education, Nanjing, China; 2 State Key Laboratory for Biology of Plant Diseases and Insect Pests, Institute of Plant Protection, Chinese Academy of Agricultural Sciences, Beijing, China; USDA-ARS Beltsville Agricultural Research Center, UNITED STATES

## Abstract

In order to acquire enough nutrients and energy for further development, larvae need to invest a large portion of their sensory equipments to identify food sources. Yet, the molecular basis of odor-driven behavior in larvae has been poorly investigated. Information on olfactory genes, particularly odorant binding proteins (OBPs) and chemosensory proteins (CSPs) which are involved in the initial steps of olfaction is very scarce. In this study, we have identified 26 OBP and 21 CSP genes from the transcriptomes of *Helicoverpa armigera* larval antennae and mouthparts. A comparison with the 34 OBP and 18 CSP genes of the adult antenna, revealed four novel OBPs and seven novel CSPs. Similarly, 27 OBPs (six novel OBPs) and 20 CSPs (6 novel CSPs) were identified in the transcriptomes of *Helicoverpa assulta* larval antennae and mouthparts. Tissue-specific profiles of these soluble proteins in *H*. *armigera* showed that 6 OBP and 4 CSP genes are larval tissue-specific, 15 OBPs and 13 CSPs are expressed in both larvae and adult, while the rest are adult- specific. Our data provide useful information for functional studies of genes involved in larval foraging.

## Introduction

For survival, insects need a specialised sensory system to monitor environmental odors. Olfactory stimuli in Lepidoptera can be divided into intra-specific pheromones, mainly mediating communication between sexes, and plant volatiles used as cues for larval foraging and oviposition [1–3]. Odor detection is achieved by ten thousand chemosensilla on the two main sensory organs, antenna and mouthparts, housing olfactory sensory neurons (OSNs) that respond to volatiles and send electrical impulses to antennal lobes. From these organs cognate project neurons (PN) convey electric signals to the mushroom bodies and lateral horn of the protocerebron, triggering behavioral responses [4–6]. At the periphery, several protein families are involved in odor detection: odorant binding proteins (OBPs), chemosensory proteins (CSP), odorant receptors (OR) and ionotropic receptors (IR) [7–9]. Of these genes, the receptor families including ORs and IRs are the key elements which determine both sensitivity and specificity of chemical recognition. ORs are seven trans-membrane domain receptors expressed in the dendrite membrane of olfactory sensory neurons. OR perform their function as heterodimer with a specific ligand-binding ORx and a highly conserved co-receptor named Orco [10–12]. IRs belong to the ionotropic glutamate receptor (iGluR)-like protein family and can be activated by small molecules like acetates and amine-like volatile compounds [13–15].

Apart from receptors, two families of soluble proteins, OBPs and CSPs, also play essential roles in the first step of olfactory detection. OBPs are small soluble proteins generally with 135–220 amino acids. To maintain a compact and conserved structure, six conserved cysteines are paired in three interlocked disulphide bridges. Six α-helices envelop a hydrophobic binding pocket [16–18]. OBPs are present at high concentrations (up to 10mM) in the lymph between the dendritic membrane and the cuticular wall [7, 19]. More interesting, there are some evidences that OBPs contribute to odorant recognition, rather than being passive odorant shuttles [20, 21]. Some studies have shown that OBPs perform the first filtering function in olfactory discrimination [19,20], besides a more general role in ferrying ligands through the sensillum lymph to the membrane of OSN dendrites. OBPs have also been shown to influence the response of ORx/ORco complexes to specific odors [21, 22]. CSPs represent another class of small soluble proteins abundant in the lymph of chemosensilla [23]. They are different from OBPs in amino acid sequence and structure, but appear to be similar in functions, although better evidence is needed to clarify their role in olfaction.

In Lepidoptera, both larvae and adults use their olfactory system to detect volatile chemicals, but their olfactory organs are completely different in morphology. In adults, a pair of antennae bear tens of thousands of sensilla, each of them housing two or more OSNs [24, 25]. Larvae are equipped with two different olfactory organs, antennae and mouthparts [26,27]. Unlike adult antennae, larval antennae and mouthparts contain few sensilla, but each of them houses a cluster of OSNs [28, 29]. With the rapid development of next generation sequence techniques, a large number of olfactory genes including IRs, ORs, OBPs and CSPs have been recently identified in the antennae of several moths, such as *Manduca sexta* [15, 30–32], *Helicoverpa armigera* [33–35], *Helicoverpa assulta* [34, 36], *Cydia pomonella* [37], *Spodoptera littoralis* [38, 39] and *Chilo suppressalis* [40], and many others. However, limited information is available for larval antennae and mouthparts.

Two *Helicoverpa* species, *H*. *armigera* and *H*. *assulta* are worldwide agricultural pests [41]. The behaviors of larvae and adults are largely triggered by olfactory stimuli. Previously, we performed a transcriptome analysis on adult antennae in both species. A total of 131 putative chemosensory unigenes were identified in *H*. *armigera* including 60 ORs, 19 IRs, 34 OBPs and18 CSPs. Similarly, in *H*. *assulta* we found 129 putative chemosensory unigenes, including 64 ORs, 19 IRs, 29 OBPs and 17 CSPs [34]. Skiri et al. (2005) have identified 65 glomeruli in each sex of *H*. *armigera* and 66 glomeruli in females of *H*. *assulta* [6], later supplemented by 15 new glomeruli in *H*. *armigera* [42]. Assuming that the number of glomeruli is equal to the number of ORs and IRs [43, 44], almost all olfactory receptors were identified in the two species. These data also agree with another study focused on both adults and larvae of *H*. *armigera* chemosensory tissues [35]. However, the repertoires of OBPs and CSPs in these two species may be incomplete by comparison with the numbers of OBPs and CSPs identified in the genome of *B*. *mori* (46 OBPs and 24 CSPs) [45]. This suggests that some OBP and CSP sources may occur in other chemosensory tissues, such as larval antennae and mouthparts. In this study, we performed a transcriptome analysis to identify OBP and CSP genes in larval chemosensory organs of *H*. *armigera* and *H*. *assulta*. Moreover, we conducted RT-PCR assays on *H*. *armigera* adult and larval olfactory organs to find OBP and CSP genes with specific expression in larval antennae or mouthparts.

## Methods

### Insect rearing

*H*. *armigera* were reared at the Institute of Plant Protection, Chinese Academy of Agricultural Sciences, Beijing, China. The *H*. *assulta* larvae were collected from the tobacco fields with the permission of the Experiment Station of Henan University of Science and Technology in Xuchang, Henan Province, China. Larvae were reared on an artificial diet and placed on a 16:8 h (light: dark) photoperiod at 27 ± 1°C, 55–65% RH. Pupae were sexed and male and female individuals were placed in separate cages for eclosion. The adults were fed on 10% honey solution. In expression profile studies, all adult tissues were collected from 3-day-old male and female moths, all larval tissues were collected from fifth instar larvae.

### RNA extraction

Fresh larval antennae and mouthparts were grinded in a liquid nitrogen cooled homogenizer, later adding 1mL of TriZol reagent (Invitrogen, Carlsbad, CA, USA) and the total RNA extraction were performed following the manufacturer’s instructions. The RNA sediment was dissolved in 20μL RNase-free water, RNA integrity was verified by gel electrophoresis. RNA quantity were measured on a Nanodrop ND-2000 spectrophotometer (NanoDrop products, Wilmington, DE, USA) and purity was verified by gel electrophoresis.

### cDNA library construction and sequencing

Five micrograms total RNA of each samples (*H*. *armigera* larval antennae, *H*. *armigera* larval mouthparts, *H*. *assulta* larval antennae, and *H*. *assulta* larval mouthparts,) was used to construct the cDNA library respectively. cDNA library construction and Illumina HiSeq 2000 (Illumina, San Diego, CA, USA) sequencing of the samples were performed at Beijing Genomics Institute (BGI, Shenzhen, China). The length of insert sequence was around 200 bp. The libraries were paired-end sequenced using PE90 strategy. The detailed procedures have been described in previous work from our laboratory [33, 34].

### Assembly and functional annotation

After removing low quality reads, trimming low quality nucleotides of both ends, trimming 3 adaptors and poly-A/T tails, the remainder raw-reads were considered as clean-reads. De novo assembly in each sample was conducted using Trinity (version 20120608). Then the unigenes derived from the Trinity outputs were clustered by TGICL [46,47]. The consensus cluster sequences and singletons make up the unigenes dataset. The annotation of unigenes were performed via a NCBI blastx against non-redundant (nr) and SwissProt database. Candidate unigenes encoding putative OBPs and CSPs, were identified according to nr and SwissProt annotation results.

### Sequence and phylogenetic analysis

The open reading frames (ORFs) of the putative chemosensory genes were predicted by using ORF finder (http://www.ncbi.nlm.nih.gov/gorf/gorf.html). Putative N-terminal signal peptides of OBPs and CSPs were predicted by Signal IP 4.0 (http://www.cbs.dtu.dk/services/SignalP/). Alignments of amino acid sequences (without signal peptides) were performed by ClustalX 2.0. The phylogenetic trees of OBPs and CSPs were constructed using MEGA5 software by the neighbor-joining method with Jones-Taylor-Thornton (JTT) model and the node support was assessed using a bootstrap procedure of 1000 replicates. The OBP data set contained OBP sequences identified in Lepidoptera (37 from *H*. *armigera*, 35 from *H*. *assulta*, 14 from *H*. *virescens*, 47 from *M*. *sexta* and 35 from *B*. *mori*. The CSP data set contained 25 sequences from *H*. *armigera*, 23 from *H*. *assulta*, 9 from *H*. *virescens*, 13 from *C*. *suppressalis*, and 16 from *B*. *mori*. The protein name and accession number of the genes used for phylogenetic tree building are listed in S1 Material.

### Expression analysis by semi-quantitative reverse transcription PCR

Semi-quantitative reverse transcription PCR was performed to compare the expression levels of candidate chemosensory genes in larval antennae, larval mouthparts, adult antennae and adult abdomen in *H*. *armigera*. Total RNA was extracted from each sample as mentioned above. Before cDNA synthesis, total RNA was treated with DNase I (Fermentas, Vilnius, Lithuania) to remove residual genomic DNA. The cDNA was synthesized from total RNA using RevertAid First Strand cDNA Synthesis Kit (Thermo Scientific, Waltham, MA, USA). Gene specific primers were designed using Primer-BLAST (http://www.ncbi.nlm.nih.gov/tools/primer-blast/) (S2 Material) and synthesized by Sangon Biotech Co., Ltd (Shanghai, China). Taq MasterMix (CWBIO, Beijing, China) was used for PCR reactions under general 3-step amplification of 94°C for 30s, 60°C for 30s, 72°C for 30s. For most chemosensory genes, the PCR cycle-numbers were 28. PCR products were run on a 2% agarose gel and verified by DNA sequencing. The experiment was repeated using two independently prepared cDNA templates.

## Results

### Illumina sequencing and functional annotation

In this study, the transcriptomes of larval antennae and mouthparts in *H*. *armigera* and *H*. *assulta* were sequenced by Illumina HiSeq 2000 platform. After filtering, 51.1 million and 45.5 million clean-reads of 4.6 and 4.1 gigabases were generated for larval antennae and mouthparts of *H*. *armigera*, respectively. Meanwhile, 50.2 million and 52.9 million clean-reads of 4.5 and 4.8 gigabases were generated for larval antennae and mouthparts of *H*. *assulta*. These clean reads were assembled into 47,331, 41,705, 57,789 and 47,423 unigenes in *H*. *armigera* larval antennae and mouthparts, and in *H*. *assulta* larval antennae and mouthparts, respectively. After clustering and merging, 39,371 unigenes consisting of 12,724 distinct clusters and 26,647 distinct singletons were obtained for *H*. *armigera* and 44,352 unigenes consisting of 11,179 distinct clusters and 33,173 distinct singletons were obtained for *H*. *assulta* (Table 1).

**Table 1:** Summary of data used for transcriptome assembly.

	Sample	Total Number	Total Length (nt)	Mean Length (nt)	N50	Total Consensus Sequences	Distinct Clusters	Distinct Singletons
***H*. *armigera***								
Contig	Harm-L-A	83,523	37,101,992	444	1247	-	-	-
	Harm-L-MP	71,965	32,582,057	453	1244	-	-	-
Unigene	Harm-L-A	47,331	43,755,425	924	2072	47,331	13,157	34,174
	Harm-L-MP	41,705	37,806,616	907	1953	41,705	11,955	29,750
	All	39,371	46,761,853	1188	2298	39,371	12,724	26,647
***H*. *assulta***								
Contig	Hass-L-A	103,673	38,454,494	371	831	-	-	-
	Hass-L-MP	78,235	36,088,209	461	1241	-	-	-
Unigene	Hass-L-A	57,789	40,537,082	701	1554	57,789	8,354	49,435
	Hass-L-MP	47,423	42,770,981	902	2028	47,423	9,677	37,746
	All	44,352	46,940,349	1058	2104	44,352	11,179	33,173

A blastx homology search against the NCBI nr protein database revealed that 22,628 (57.5%) and 22,724 (51.2%) unigenes from *H*. *armigera* and *H*. *assulta*, respectively, showed sequence similarities to known proteins, with a cut-off E-value of 10^−5^. In the nr homologous species distribution, 46.78% (*H*. *armigera*) and 48.42% (*H*. *assulta*) annotated sequences closely matched the sequences of *B*. *mori*. The next most similar species was *D*. *plexippus* whose sequences matched 26.25% of those of *H*. *armigera* and 27.13% of *H*. *assulta*. Only a low percentage (<5%) of *H*. *armigera* and *H*. *assulta* sequences had orthologues in other species (S3 Material).

### Identification of putative odorant-binding proteins

Based on the blastx sequence homology searching, a total of 26 and 27 OBP genes were obtained from *H*. *armigera* and *H*. *assulta* larval transcriptome respectively. Of these genes, 22 HarmOBPs and HassOBPs presented intact ORFs encoding for proteins of 135 to 195 aa, all exhibiting signal peptides at their N-termini (Table 2). A comparison with known OBPs of *H*. *armigera* adult antenna revealed four novel OBPs in larvae, that we named as HarmOBP31, HarmOBP33, HarmOBP35 and HarmOBP36, and six new OBPs in *H*. *assulta*, named as HassOBP33 to HassOBP38. All these novel genes were deposited in the GeneBank databases with the following accession numbers: HarmOBP31: KY810175, HarmOBP33: KY810179, HarmOBP35: KY810176, HarmOBP36: KY810177, HassOBP33: KY810180, HassOBP34: KY810178, HassOBP35: KY810181, HassOBP36: KY810182, HassOBP37: KY810183, HassOBP38: KY815028.

**Table 2:** Unigenes of candidate odorant binding proteins in larval chemosensory tissues of *H*. *armigera* and *H*. *assulta*.

Unigene reference	Gene name	Length (bp)	ORF (aa)	Blastx best hit (Reference/Name/Species)	E value	Identity	Signal peptide	Full length
***H*. *armigera***								
Unigene16494	HarmGOBP2	634	162	emb|CAC08211.1|general odorant-binding protein 2 precursor (GOBP2) [Helicoverpa armigera]	6E-115	100%	Yes	Yes
Unigene15587	HarmOBP1	617	147	gb|AEX07272.1|odorant-binding protein [Helicoverpa assulta]	1E-80	94%	Yes	Yes
Unigene25962	HarmOBP2	276	51	gb|AGH70103.1|odorant binding protein 7 [Spodoptera exigua]	4E-22	84%	Yes	No
Unigene8467	HarmOBP3	662	147	gb|AGC92788.1|odorant-binding protein 3 [Helicoverpa assulta]	2E-97	96%	Yes	Yes
Unigene3463	HarmOBP4	611	147	gb|AEX07276.1|odorant-binding protein [Helicoverpa assulta]	2E-89	91%	Yes	Yes
CL5000.Contig1	HarmOBP5	641	147	gb|AEX07271.1|odorant-binding protein [Helicoverpa assulta]	1E-101	99%	Yes	Yes
CL1168.Contig2	HarmOBP6	641	147	gb|AEX07270.1|odorant-binding protein [Helicoverpa assulta]	2E-98	97%	Yes	Yes
Unigene9848	HarmOBP9	703	148	gb|AGC92789.1|odorant-binding protein 9 [Helicoverpa assulta]	7E-106	99%	Yes	Yes
CL3933.Contig2	HarmOBP14	769	137	gb|AFI57167.1|odorant-binding protein 18 [Helicoverpa armigera]	1E-94	100%	Yes	Yes
CL3679.Contig2	HarmOBP15	631	168	gb|ADY17882.1|odorant binding protein [Spodoptera exigua]	6E-82	75%	Yes	Yes
Unigene12555	HarmOBP16	714	186	gb|AEX07273.1|odorant-binding protein [Helicoverpa assulta]	3E-78	62%	Yes	Yes
Unigene9920	HarmOBP17	473	137	gb|AGM38607.1|odorant binding protein [Chilo suppressalis]	1E-58	69%	Yes	Yes
Unigene371	HarmOBP19	533	148	ref|NP_001140188.1|odorant-binding protein 4 [Bombyx mori]	3E-37	46%	Yes	Yes
Unigene16501	HarmOBP21	550	142	gb|AFD34178.1|odorant binding protein 2 [Argyresthia conjugella]	3E-45	54%	Yes	Yes
Unigene24118	HarmOBP22	576	140	gb|AFG72998.1|odorant-binding protein 1 [Cnaphalocrocis medinalis]	3E-55	57%	Yes	No
Unigene3491	HarmOBP23	811	241	gb|AGH70107.1|odorant binding protein 11 [Spodoptera exigua]	3E-95	80%	Yes	No
Unigene10971	HarmOBP25	658	195	gb|AEX07273.1|odorant-binding protein [Helicoverpa assulta]	1E-136	98%	Yes	Yes
Unigene14030	HarmOBP26	669	154	gb|EHJ67765.1|odorant binding protein [Danaus plexippus]	1E-60	69%	Yes	Yes
CL376.Contig1	HarmOBP27	577	147	gb|AEX07279.1|odorant-binding protein [Helicoverpa armigera]	7E-97	96%	Yes	Yes
Unigene15643	HarmOBP28	643	147	dbj|BAI44700.1|odorant binding protein [Bombyx mori]	6E-52	56%	Yes	Yes
Unigene6228	HarmOBP29	608	142	gb|AAR28763.1|odorant-binding protein-2 precursor [Spodoptera frugiperda]	1E-46	62%	Yes	Yes
Unigene8997	HarmOBP30	794	135	gb|AFI57166.1|odorant-binding protein 17 [Helicoverpa armigera]	1E-93	99%	Yes	Yes
Unigene9430	HarmOBP31	755	150	gb|AEX07271.1|odorant-binding protein [Helicoverpa assulta]	2E-59	61%	Yes	Yes
Unigene5227	HarmOBP33	351	99	ref|XP_004928233.1|general odorant-binding protein 99a-like [Bombyx mori]	1E-41	65%	No	No
Unigene6209	HarmOBP35	656	146	gb|AFI57165.1|odorant-binding protein 16 [Helicoverpa armigera]	5E-108	99%	Yes	Yes
Unigene7375	HarmOBP36	537	149	ref|NP_001140188.1|odorant-binding protein 4 [Bombyx mori]	4E-40	45%	Yes	Yes
***H*. *assulta***								
Unigene23306	HassGOBP1	328	109	sp|Q27226.1|general odorant binding protein 1 [Heliothis virescens]	9E-73	96%	Yes	No
Unigene21063	HassGOBP2	624	162	gb|AAQ54909.1|general odorant binding protein 2 [Helicoverpa assulta]	8E-115	100%	Yes	Yes
CL3828.Contig1	HassOBP1	1042	147	gb|AEX07272.1|odorant-binding protein [Helicoverpa assulta]	1E-81	98%	Yes	Yes
CL2155.Contig1	HassOBP2	752	143	gb|AGH70103.1|odorant binding protein 7 [Spodoptera exigua]	7E-85	82%	Yes	Yes
Unigene16541	HassOBP3	618	147	gb|AGC92788.1|odorant-binding protein 3 [Helicoverpa assulta]	2E-100	100%	Yes	Yes
Unigene8150	HassOBP4	581	147	gb|AEX07276.1|odorant-binding protein [Helicoverpa assulta]	2E-96	97%	Yes	Yes
Unigene6153	HassOBP5	637	147	gb|AEX07271.1|odorant-binding protein [Helicoverpa assulta]	1E-101	99%	Yes	Yes
Unigene5533	HassOBP6	626	147	gb|AEX07270.1|odorant-binding protein [Helicoverpa assulta]	2E-101	99%	Yes	Yes
Unigene8860	HassOBP9.2	698	148	gb|AGC92789.1|odorant-binding protein 9 [Helicoverpa assulta]	5E-105	99%	Yes	Yes
Unigene18089	HassOBP14	1747	137	gb|AFI57167.1|odorant-binding protein 18 [Helicoverpa armigera]	1E-89	99%	Yes	Yes
Unigene18604	HassOBP15	579	166	gb|ADY17882.1|odorant binding protein [Spodoptera exigua]	1E-82	76%	Yes	No
Unigene4097	HassOBP19	516	148	ref|NP_001140188.1|odorant-binding protein 4 [Bombyx mori]	1E-37	47%	Yes	Yes
Unigene5122	HassOBP22	379	125	gb|AGM38613.1|odorant binding protein [Chilo suppressalis]	2E-54	58%	No	No
Unigene1471	HassOBP23	863	241	gb|AGH70107.1|odorant binding protein 11 [Spodoptera exigua]	8E-96	81%	Yes	No
Unigene12884	HassOBP25	621	194	gb|AEX07273.1|odorant-binding protein [Helicoverpa assulta]	8E-115	100%	Yes	Yes
Unigene8198	HassOBP26	669	181	gb|EHJ67765.1|odorant binding protein [Danaus plexippus]	9E-73	96%	Yes	No
CL3623.Contig1	HassOBP27	573	147	gb|AEX07279.1|odorant-binding protein [Helicoverpa armigera]	1E-22	48%	No	No
Unigene6098	HassOBP28	585	147	dbj|BAI44700.1|odorant binding protein [Bombyx mori]	1E-37	47%	Yes	Yes
Unigene6100	HassOBP29	602	142	gb|AAR28763.1|odorant-binding protein 2 precursor [Spodoptera frugiperda]	2E-54	58%	No	No
Unigene11827	HassOBP30	811	135	gb|AFI57166.1|odorant-binding protein 17 [Helicoverpa armigera]	8E-96	81%	Yes	No
Unigene6144	HassOBP31	789	150	gb|AEX07271.1|odorant-binding protein [Helicoverpa assulta]	5E-134	97%	Yes	Yes
Unigene10157	HassOBP33	609	152	ref|XP_004928233.1|general odorant-binding protein 99a-like [Bombyx mori]	1E-60	69%	Yes	Yes
Unigene13394	HassOBP34	476	137	gb|AGM38607.1|odorant binding protein [Chilo suppressalis]	8E-98	97%	Yes	Yes
Unigene16490	HassOBP35	636	146	gb|AGC92791.1|odorant-binding protein 16 [Helicoverpa assulta]	3E-52	56%	Yes	Yes
Unigene2048	HassOBP36	527	149	ref|NP_001140188.1|odorant-binding protein 4 [Bombyx mori]	8E-45	60%	Yes	Yes
Unigene20923	HassOBP37	634	142	gb|AFD34178.1|odorant binding protein 2 [Argyresthia conjugella]	1E-93	99%	Yes	Yes
Unigene31389	HassOBP38	356	118	gb|AGC92793.1|odorant-binding protein 19 [Helicoverpa assulta]	4E-59	62%	Yes	Yes

**Fig 1:**
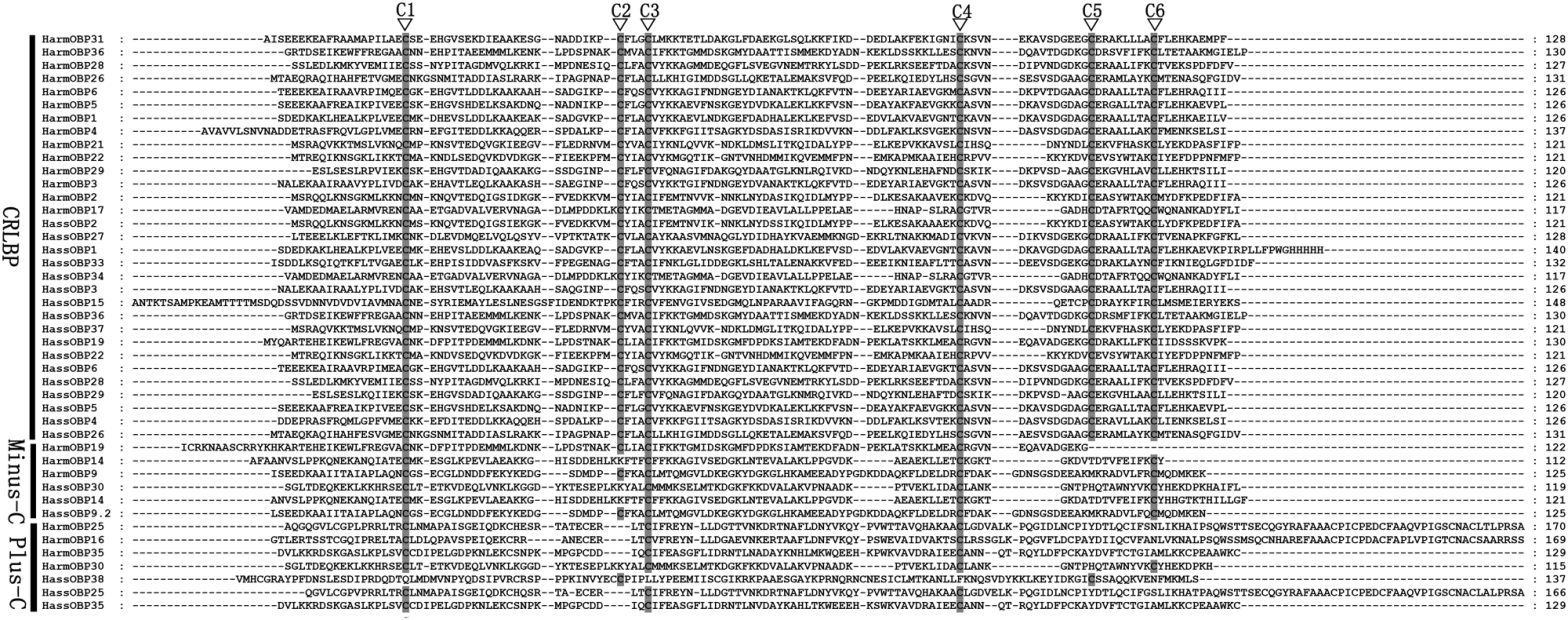
Sequences alignment of candidate HarmOBPs and HassOBPs. The conserved cysteine residues were marked with “▽”. All these OBPs were assignment into CRLBP with six conserved cysteine residues, Minus-C with four conserved cysteine residues and Plus-C with more than more than six conserved cysteine residues.

Insect OBPs are generally grouped into three main subfamilies: “Classic” OBPs with six conserved cysteines, “Minus-C” with only four cysteines, and “Plus-C” with more cysteines in addition to those of the conserved motif [45, 48, 49]. Among the larval OBPs, 14 of *H*. *armigera* and 17 of *H*. *assulta* were assigned to the Classic OBP group, while 3 can be classified as Minus-C OBPs in both species. 7 OBPs in both species belong to the Plus-C group, while others could not be assigned due to incomplete sequences (Fig 1).

**Fig 2:**
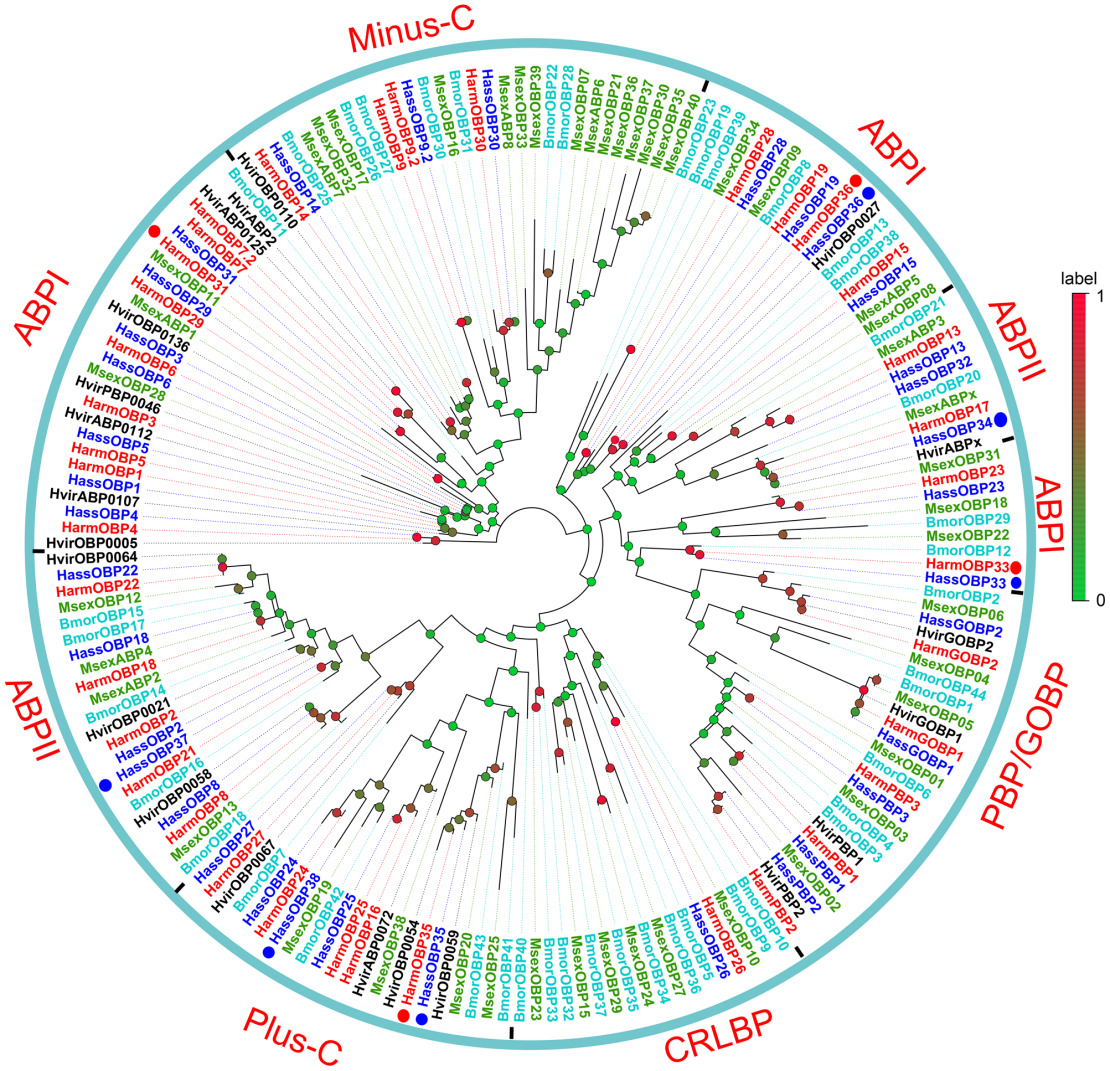
Phylogenetic tree of OBPs from *H*. *armigera*, *H*. *assulta* and other Lepidoptera insects. Harm: *H*. *armigera* (red), Hass: *H*. *assulta* (blue), Hvir: *H*. *virescens* (black), Bm: *B*. *mori* (aquamarine), Msex: *M*. *sexta* (cyan) The whole tree can be divided into six branches including GOBP/PBP, CRLBP, Plus-C, Minus-C, ABPI and ABPII. The red and blue circles represented newly identified HarmOBPs and HassOBPs respectively.

A phylogenetic tree was constructed using OBP sequences from *H*. *armigera*, *H*. *assulta*, *H*. *virescens*, *M sexta* and *B*. *mori* (Fig 2). Accordingly, the OBPs can be grouped into ABPI (antennal binding protein I), ABPII (antennal binding protein II), CRLBP (classic OBP), Minus-C, Plus-C, and PBP/GOBP (general odorant binding protein/pheromone binding protein) clusters based on the classification of OBPs from *B*. *mori* [45]. At the same time, most OBPs of *H*. *armigera* and *H*. *assulta* defined as Minus-C and Plus-C clustered with *B*. *mori* proteins of the same groups. However, among “classic” OBPs, only two sequences were found in the CRLBP branch, the others in the ABPX branches. Based on the bootstrap values on the tree, for all novel HarmOBPs we could find orthologous genes in *H*. *assulta* with more than 90% sequence identity. Only for HassOBP38 we could not identify an orthologue in *H*. *armigera*.

### Identification of candidate chemosensory proteins

**Fig 3:**
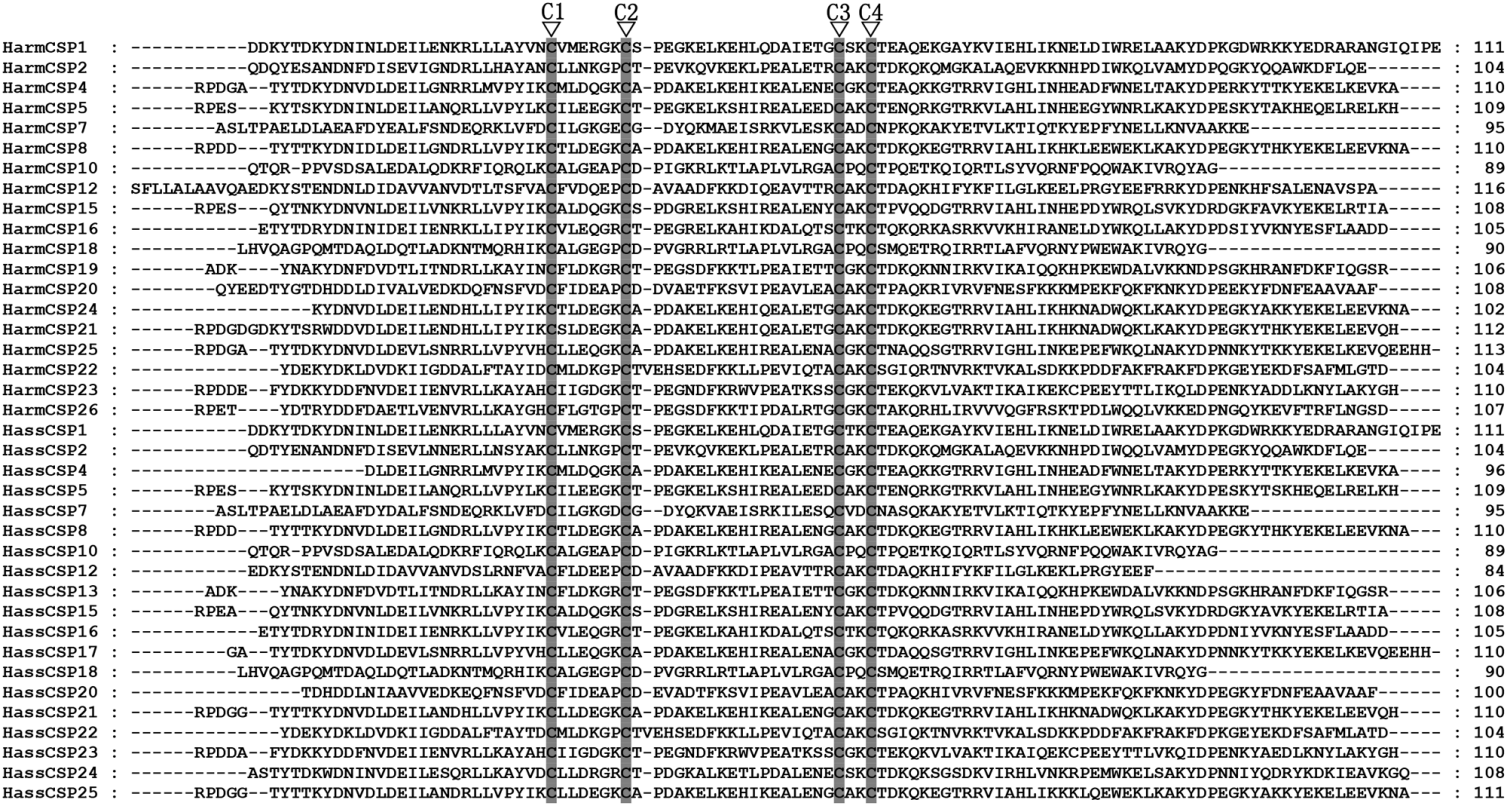
Sequences alignment of candidate HarmCSPs and HassCSPs. All these CSPs were characteristic of four conserved cysteine residues marked with “▽”.

**Fig 4:**
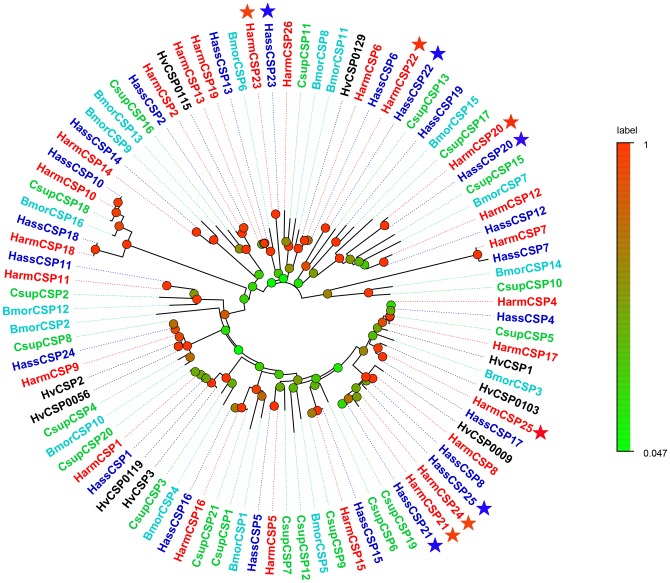
Phylogenetic tree of CSPs *H*. *armigera*, *H*. *assulta* and other Lepidoptera insects. Harm: *H*. *armigera* (red), Hass: *H*. *assulta* (blue), Hvir: *H*. *virescens* (black), Bm: *B*. *mori* (aquamarine), Csup: *C*. *suppressalis* (cyan). The red and blue pentastars represented newly identified HarmCSPs and HassCSPs respectively.

In our transcription sets, a total of 21 sequences in *H*. *armigera* and 20 sequences in *H*. *assulta* can be matched with sequences of known CSPs in other Lepidoptera species. Of these, 17 HarmCSPs and HassCSPs had full-length ORFs and predicted signal peptides. Their lengths range from 107 to 292 amino acids (Table 3). A comparison with CSPs previously reported for *H*. *armigera* and *H*. *assulta* adult antenna revealed seven new sequences in *H*. *armigera* (HarmCSP20 to HarmCSP26) and six in *H*. *assulta* (HassCSP20 to HassCSP25). All candidate CSPs exhibit the four conversed cysteine pattern characteristic of this family (Fig 3). These sequences were used to build a neighbor-joining tree with the CSPs of *C*. *suppressalis*, *B*. *mori* and *H*. *virescens*. In the tree we could recognize four groups of genes clustered together with a 99% bootstrap value, while the remaining sequences could not be grouped. Based on this homology analysis, we named the novel CSPs as HarmCSP20/HassCSP20, HarmCSP21/HassCSP21, HarmCSP22/HassOBP22, HarmCSP23/HassCSP23, HarmCSP24, HarmCSP25, HarmCSP26, HassCSP24 and HassCSP25 following the numbers assigned to previously reported CSPs (Fig 4). All these novel genes were deposited in the GeneBank: HarmCSP20-26 (GeneBank accession numbers: KY810184, KY810185, KY810186, KY810187, KY810188, KY815026, KY815027), HassCSP20-25 (GeneBank accession numbers: KY810189, KY810190, KY810191, KY810192, KY810193, KY810194).

**Table 3:** Unigenes of candidate chemosensory proteins in larval chemosensory tissues of *H*. *armigera* and *H*. *assulta*.

Unigene reference	Gene name	Length (bp)	ORF (aa)	Blastx best hit (Reference/Name/Species)	E value	Identity	Signal peptide	Full length
***H*. *armigera***								
Unigene12801	HarmCSP1	745	127	gb|ABB91378.1|chemosensory protein [Helicoverpa assulta]	1.00E-87	99%	Yes	Yes
Unigene12890	HarmCSP2	523	120	gb|AGR39574.1|chemosensory protein 4 [Agrotis ipsilon]	1.00E-73	86%	Yes	Yes
Unigene2914	HarmCSP4	381	96	gb|AFR92093.1|chemosensory protein 9 [Helicoverpa armigera]	2.00E-65	98%	No	No
Unigene4249	HarmCSP5	730	127	gb|AGY49267.1|putative chemosensory protein [Sesamia inferens]	4.00E-65	71%	Yes	Yes
Unigene4261	HarmCSP7	684	111	gb|AGY49261.1|putative chemosensory protein [Sesamia inferens]	1.00E-47	61%	Yes	Yes
CL750.Contig1	HarmCSP8	546	128	gb|AFR92095.1|chemosensory protein 11 [Helicoverpa armigera]	9.00E-91	99%	Yes	Yes
Unigene8213	HarmCSP9	389	99	gb|AGH20055.1|chemosensory protein 17, partial [Helicoverpa armigera]	2.00E-68	100%	No	No
Unigene2863	HarmCSP10	1000	107	gb|AGY49264.1|putative chemosensory protein [Sesamia inferens]	2.00E-64	92%	Yes	Yes
Unigene6030	HarmCSP12	377	103	gb|AFR92092.1|chemosensory protein 8 [Helicoverpa armigera]	1.00E-68	100%	Yes	No
CL1562.Contig1	HarmCSP14	2323	292	ref|NP_001037069.1|chemosensory protein 9 precursor [Bombyx mori]	1.00E-81	70%	Yes	Yes
Unigene8181	HarmCSP15	516	124	gb|AGH20053.1|chemosensory protein 15, partial [Helicoverpa armigera]	1.00E-75	99%	Yes	Yes
CL5163.Contig1	HarmCSP16	544	123	gb|AGR39578.1|chemosensory protein 8 [Agrotis ipsilon]	1.00E-66	76%	Yes	Yes
CL513.Contig1	HarmCSP18	864	107	gb|AGY49260.1|putative chemosensory protein, partial [Sesamia inferens]	1.00E-45	99%	Yes	Yes
Unigene2882	HarmCSP19	1216	122	gb|AFR92094.1|chemosensory protein 10 [Helicoverpa armigera]	3.00E-25	42%	Yes	Yes
CL1091.Contig1	HarmCSP20	1056	127	gb|AGH20054.1|chemosensory protein 16, partial [Helicoverpa armigera]	8.00E-75	93%	Yes	Yes
CL750.Contig3	HarmCSP21	567	130	gb|AFR92098.1|chemosensory protein 14 [Helicoverpa armigera]	4.00E-85	93%	Yes	Yes
Unigene14000	HarmCSP22	545	122	dbj|BAG71920.1|chemosensory protein 12 [Papilio xuthus]	2.00E-43	58%	Yes	Yes
Unigene7878	HarmCSP23	507	125	ref|NP_001037066.1|chemosensory protein precursor [Bombyx mori]	2.00E-49	60%	Yes	Yes
CL750.Contig2	HarmCSP24	404	102	gb|AFR92098.1|chemosensory protein 14 [Helicoverpa armigera]	3.00E-64	91%	No	No
Unigene12750	HarmCSP25	577	131	gb|AFR92093.1|chemosensory protein 9 [Helicoverpa armigera]	5.00E-74	82%	Yes	Yes
Unigene4081	HarmCSP26	458	123	gb|AIW65100.1| chemosensory protein [Helicoverpa armigera]	5E-85	99%	Yes	Yes
***H*. *assulta***								
Unigene15996	HassCSP1	746	127	gb|ABB91378.1|chemosensory protein [Helicoverpa assulta]	1.00E-86	98%	Yes	Yes
Unigene20555	HassCSP2	530	120	gb|AGR39574.1|chemosensory protein 4 [Agrotis ipsilon]	1.00E-73	94%	Yes	Yes
CL2298.Contig1	HassCSP4	374	96	gb|AFR92093.1|chemosensory protein 9 [Helicoverpa armigera]	6.00E-65	99%	No	No
CL332.Contig1	HassCSP5	693	127	gb|AGY49267.1|putative chemosensory protein [Sesamia inferens]	9.00E-66	88%	Yes	Yes
CL3839.Contig2	HassCSP7	542	111	gb|AGY49261.1|putative chemosensory protein [Sesamia inferens]	1.00E-47	86%	Yes	Yes
CL2298.Contig2	HassCSP8	504	129	gb|AFR92095.1|chemosensory protein 11 [Helicoverpa armigera]	1.00E-85	98%	Yes	Yes
Unigene3452	HassCSP10	1081	107	gb|AGY49264.1|putative chemosensory protein [Sesamia inferens]	4.00E-64	95%	Yes	Yes
Unigene3770	HassCSP12	449	103	gb|AFR92092.1|chemosensory protein 8 [Helicoverpa armigera]	1.00E-63	98%	Yes	No
Unigene20371	HassCSP13	1220	122	gb|AFR92094.1|chemosensory protein 10 [Helicoverpa armigera]	1.00E-24	63%	Yes	Yes
CL3062.Contig3	HassCSP14	1951	292	ref|NP_001037069.1|chemosensory protein 9 precursor [Bombyx mori]	1.00E-83	81%	Yes	Yes
Unigene20571	HassCSP15	330	84	gb|AGH20053.1|chemosensory protein 15, partial [Helicoverpa armigera]	1.00E-57	100%	No	No
Unigene20462	HassCSP16	550	123	gb|AGR39578.1|chemosensory protein 8 [Agrotis ipsilon]	5.00E-67	89%	Yes	Yes
Unigene2580	HassCSP17	597	131	gb|AFR92093.1|chemosensory protein 9 [Helicoverpa armigera]	2.00E-74	91%	Yes	Yes
Unigene16273	HassCSP18	385	107	gb|AGY49260.1|putative chemosensory protein, partial [Sesamia inferens]	6.00E-48	99%	Yes	Yes
CL953.Contig1	HassCSP20	883	122	gb|AGH20054.1|chemosensory protein 16, partial [Helicoverpa armigera]	3.00E-77	98%	Yes	Yes
CL2298.Contig3	HassCSP21	528	128	gb|AFR92095.1|chemosensory protein 11 [Helicoverpa armigera]	1.00E-89	99%	Yes	Yes
Unigene20634	HassCSP22	537	122	dbj|BAG71920.1|chemosensory protein 12 [Papilio xuthus]	1.00E-43	75%	Yes	Yes
Unigene12407	HassCSP23	488	125	ref|NP_001037066.1|chemosensory protein precursor [Bombyx mori]	6.00E-49	77%	Yes	Yes
Unigene10302	HassCSP24	595	126	gb|AAM77040.1|chemosensory protein 2 [Heliothis virescens]	1.00E-82	98%	Yes	Yes
Unigene12416	HassCSP25	539	129	gb|AFR92095.1|chemosensory protein 11 [Helicoverpa armigera]	6.00E-82	96%	Yes	Yes

### Expression of the OBPs and CSPs in larva and adult *H*. *armigera*

**Fig 5:**
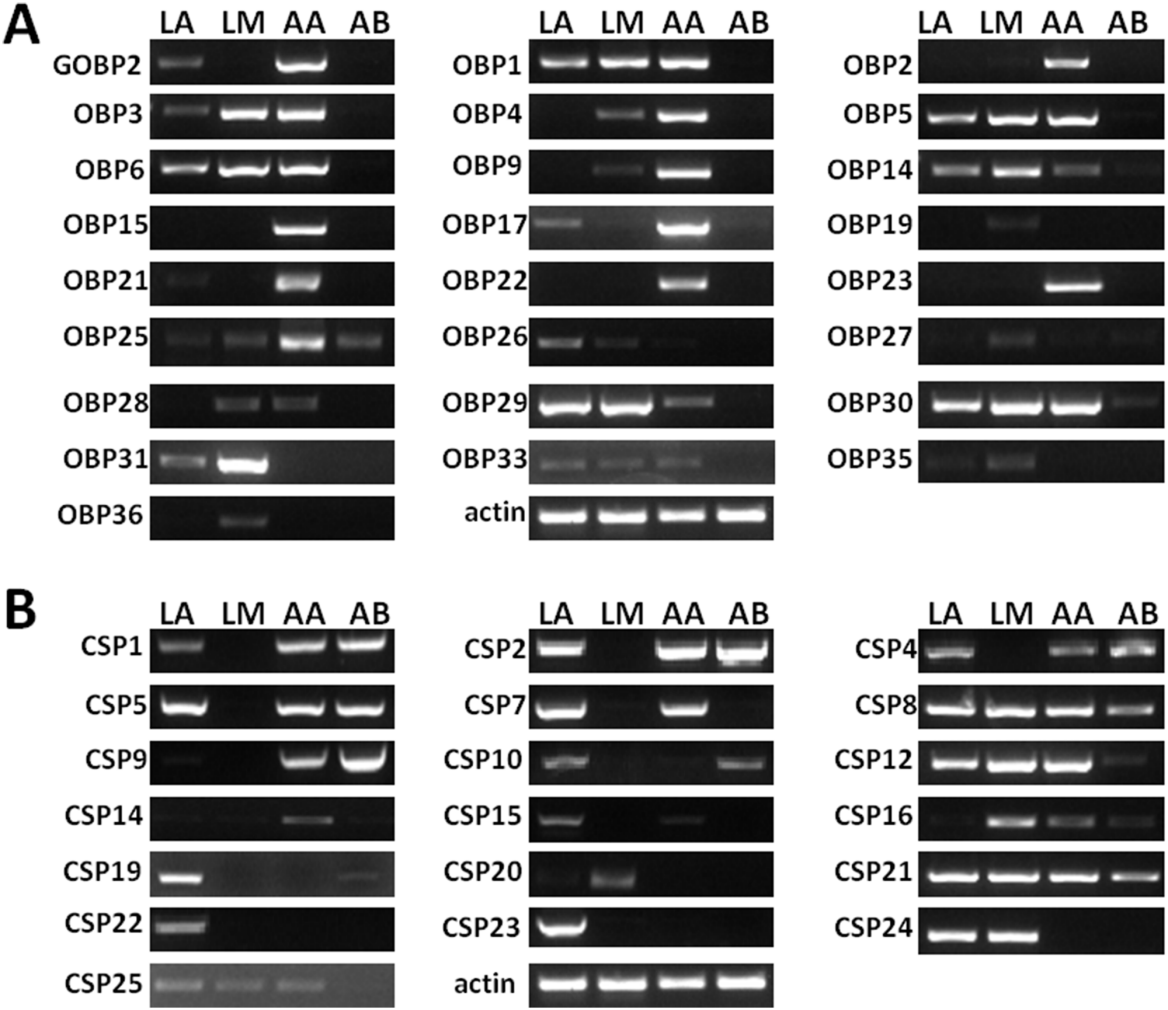
Expression of candidate OBPs and CSPs in larva and adult *H*. *armigera*. LA: larval antenna; LM: larval mouthparts; AA: adult antenna; AB: adult abdomen.

To better understand the functional role of OBPs and CSPs in larval olfactory systems, we investigated the expression patterns of all candidate HarmOBPs and HarmCSPs via semi-quantitative reverse transcription PCR. The tissues used were larval antenna, larval mouthpart, adult antenna and adult abdomen. The results reported in Fig 5 show that all OBPs except HarmOBP16 were successfully detected in target tissues. Six OBPs were exclusive to larval tissues including HarmOBP36, HarmOBP27 and HarmOBP19 specific for larval mouthparts, while HarmOBP26, HarmOBP31 and HarmOBP35 were expressed in both larval antennae and mouthparts. On the other hand, we found that five OBPs (HarmOBP2, HarmOBP15 and HarmOBP21, HarmOBP22 and HarmOBP23) are exclusively expressed in adult antenna. The remaining 13 OBPs showed expression in both larval and adult tissues. Of this latter group HarmGOBP2, HarmOBP4, HarmOBP9, HarmOBP17 and HarmOBP25 where were preferentially expressed in adult antenna, while the others did not show significant differences between larval and adult tissues. Compared to OBPs, CSPs were more expressed in non-olfactory tissues suggesting diverse functions. Eight of them showed similar expression levels in all tissues, while the others were specifically detected in olfactory organs. In particular, four genes (HarmCSP20, 22, 23 and 24) were specific of larval olfactory tissues, one (HarmCSP14) was detected only in adult antenna, and three (HarmCSP7, HarmCSP15 and HarmCSP25) were found in both larval and adult olfactory organs with no significant differences.

## Discussion

In Lepidoptera, the main tasks of adults are reproduction and species dispersal. To accomplish them they use a sophisticated olfactory system for correct mating and oviposition on the suitable host plant [50, 51]. Compared to adults, larvae show limited activity, their major tasks being feeding, growing and accumulating energy [52,53]. Therefore larvae are expected to harbor a simpler olfactory system than adults. One of the characteristics of monophagous insects is the strict specificity to their host, a typical example being the specialization of *M*. *sexta* for Solanaceous plants [32]. In this case, the mother choses the host plant while ovipositing and larvae may not need to move away through their life [54, 55]. In contrast, larvae of polyphagous speciesoften ignore their mother’s choices, disperse actively, and often move between different host plants for feeding [56, 57]. For example, sometimes larvae need to abandon their prior host and select another one, because the plant resources are exhausted, or because of competition with other herbivores, or else because the plant has become infected [58–61]. Such differences in foraging behaviors are genetically determined [56].

*H*. *armigera* and *H*. *assulta* are two closely related species both representing serious pests in China and other countries. *H*. *armigera* is a polyphagous insect which attacks about 180 species of plants [62], while *H*. *assulta* is oligophagous, mainly feeding on tobacco [63]. In both species antennae and mouthparts are the main chemosensory structures guiding the larvae to their host plants. Thus, a study of larval antennae and mouthparts at the molecular level can provide useful information for larva-based pest control.

In this work, we focused on two families of soluble protein OBPs and CSPs which play some roles in the interactions between odorant molecules and olfactory receptors. We identified a total of 26 OBPs and 21 CSPs in the larval chemosensory tissues of *H*. *armigera* as well as 27 OBPs and 20 CSPs in *H*. *assulta*. Combined with the data available for in adult antennae, the total number of OBP genes identified in *H*. *armigera* and *H*. *assulta* are 38 and 35 respectively. These numbers are lower, although in the same order, than those reported for other species (46 in *B*. *mori*) [45]. The total number of CSP genes identified in *H*. *armigera* (25) and *H*. *assulta* (23) are also in the same order of magnitude as in other species such as *B*. *mori* (21), and *S*. *littoralis* (23) [39, 45].

For most of HarmOBPs and HarmCSPs we could find homologue genes in *H*. *assulta*. The high similarities in sequence between pairs of orthologous genes suggest that *H*. *armigera* and *H*. *assulta* larvae detect similar volatile substances. This idea is supported by the observation that often mixed populations of the two species are present on tobacco and some solanaceous plants [63]. However, for some genes we could not find orthologs in the sister species. This fact, if confirmed, could suggest that during evolution, the two species can have developed some unique characteristics in their chemosensory systems to become adapted to different ecosystems. For nearly half of the HarmCSPs, we detected expression in non-olfactory organ, such as adult abdomen, suggesting roles different from chemosensing. Similarly, in other species, some CSPs were found to be expressed in non-olfactory tissues, such as the pheromone glands, where they likely assist delivery of semiochemicals in the environment [64–67], or in reproductive organs, with putative roles in egg and embryo development [68, 69]. Most of OBPs and CSPs are expressed both in adults and in larvae chemosensory organs, suggesting some common olfactory related behaviors. In particular, the gene encoding GOBP2 is expressed in larval antenna, where it might bind pheromone cues. Such hypothesis was originated from what was observed in *Plutella xylostella* [70]. However, for all PBP genes we could not find their expression in *H*. *armigera* larval tissues. This case, although being inconsistent with what was observed in *S*. *littoralis* [53], was common in other species. We also found three OBPs and six CSPs presenting larva-specific expression, suggesting that they may be involved in larval-foraging behaviors. Three OBPs and ten CSPs were found to be expressed more in larval antennae than in mouthparts, whereas the other proteins were only detected in larval mouthparts, suggesting that these genes may be involved in taste.

Our results contribute to a better understanding of the chemoreception mechanisms of larvae at the molecular level and might help the development of larva-targeted strategies for population control in these two important agricultural pests.

## Supporting information

S1 Material Accession numbers for amino acid sequences of OBPs and CSPs used in phylogenetic analyses. (DOCX)

S2 Material Primers for RT-PCR expression analyses of *H*. *armigera* OBPs and CSPs. (DOCX)

S3 Material Species distribution of unigenes’ best-hit annotation term in nr database. (A) *H*. *armigera* unigenes. (B) *H*. *assulta* unigenes.
(TIF)
