# Supplementary material for: Candidate odorant binding proteins and chemosensory proteins in the larval chemosensory tissues of two closely related noctuidae moths, Helicoverpa armigera and H. assulta
Source: PLoS One. 2017 Jun 8;12(6):e0179243. doi: 10.1371/journal.pone.0179243 (PMC5464669; doi:10.1371/journal.pone.0179243)
Supplement: S1 Material — (DOCX) [file pone.0179243.s001.docx]

**S1 material. Accession numbers for amino acid sequences of OBPs and CSPs used in phylogenetic analyses.**

**OBPs**

| **Protein name** | **Accession number** | **Protein name** | **Accession number** |
| --- | --- | --- | --- |
| HarmPBP1 | AEB54585 | BmorOBP33 | Gong et al. 2009 |
| HarmPBP2 | AEB54583 | BmorOBP34 | Gong et al. 2009 |
| HarmPBP3 | AAO16091 | BmorOBP36 | Gong et al. 2009 |
| HarmGOBP1 | AAL09821 | BmorOBP37 | Gong et al. 2009 |
| HarmOBP7 | AEB54591 | BmorOBP38 | Gong et al. 2009 |
| HarmOBP7.2 | Zhang et al. 2015 | BmorOBP39 | Gong et al. 2009 |
| HarmOBP8 | AEB54589 | BmorOBP40 | Gong et al. 2009 |
| HarmOBP9.2 | Zhang et al. 2015 | BmorOBP41 | Gong et al. 2009 |
| HarmOBP13 | AEB54584 | BmorOBP42 | Gong et al. 2009 |
| HarmOBP18 | Zhang et al. 2015 | BmorOBP43 | Gong et al. 2009 |
| HarmOBP24 | Zhang et al. 2015 | BmorOBP44 | Gong et al. 2009 |
| HassPBP1 | Zhang et al. 2015 | MsexOBP09 | Richard et al. 2015 |
| HassPBP2 | Zhang et al. 2015 | MsexOBP10 | Richard et al. 2015 |
| HassPBP3 | Zhang et al. 2015 | MsexABP1 | Richard et al. 2015 |
| HassOBP8 | Zhang et al. 2015 | MsexOBP11 | Richard et al. 2015 |
| HassOBP13 | Zhang et al. 2015 | MsexABP2 | Richard et al. 2015 |
| HassOBP18 | Zhang et al. 2015 | MsexOBP12 | Richard et al. 2015 |
| HassOBP24 | Zhang et al. 2015 | MsexABP4 | Richard et al. 2015 |
| HassOBP26 | Zhang et al. 2015 | MsexOBP13 | Richard et al. 2015 |
| HassOBP32 | Zhang et al. 2015 | MsexOBP15 | Richard et al. 2015 |
| HvirGOBP2 | CAA65606 | MsexOBP16 | Richard et al. 2015 |
| HvirABP0107 | ACX53792 | MsexABP8 | Richard et al. 2015 |
| HvirOBP0021 | ACX53711 | MsexABPx | Richard et al. 2015 |
| HvirPBP0046 | ACX53735 | MsexOBP18 | Richard et al. 2015 |
| HvirOBP0005 | ACX53696 | MsexOBP20 | Richard et al. 2015 |
| HvirABP0112 | ACX53797 | MsexOBP19 | Richard et al. 2015 |
| HvirOBP0136 | ACX53819 | MsexOBP38 | Richard et al. 2015 |
| HvirOBP0058 | ACX53747 | MsexOBP21 | Richard et al. 2015 |
| HvirABP2 | CAC33574 | MsexOBP22 | Richard et al. 2015 |
| HvirOBP0110 | ACX53795 | MsexOBP23 | Richard et al. 2015 |
| HvirOBP0072 | ACX53761 | MsexOBP24 | Richard et al. 2015 |
| HvirOBP0054 | ACX53743 | MsexOBP07 | Richard et al. 2015 |
| HvirOBP0067 | ACX53756 | MsexABP6 | Richard et al. 2015 |
| HvirABPX | CAA05508 | MsexOBP17 | Richard et al. 2015 |
| BmorOBP1 | Gong et al. 2009 | MsexOBP33 | Richard et al. 2015 |
| BmorOBP2 | Gong et al. 2009 | MsexABP7 | Richard et al. 2015 |
| BmorOBP3 | Gong et al. 2009 | MsexOBP32 | Richard et al. 2015 |
| BmorOBP4 | Gong et al. 2009 | MsexOBP06 | Richard et al. 2015 |
| BmorOBP6 | Gong et al. 2009 | MsexOBP03 | Richard et al. 2015 |
| BmorOBP7 | Gong et al. 2009 | MsexOBP01 | Richard et al. 2015 |
| BmorOBP8 | Gong et al. 2009 | MsexOBP04 | Richard et al. 2015 |
| BmorOBP11 | Gong et al. 2009 | MsexOBP02 | Richard et al. 2015 |
| BmorOBP13 | Gong et al. 2009 | MsexOBP25 | Richard et al. 2015 |
| BmorOBP15 | Gong et al. 2009 | MsexOBP05 | Richard et al. 2015 |
| BmorOBP17 | Gong et al. 2009 | MsexABP5 | Richard et al. 2015 |
| BmorOBP18 | Gong et al. 2009 | MsexOBP27 | Richard et al. 2015 |
| BmorOBP20 | Gong et al. 2009 | MsexABP3 | Richard et al. 2015 |
| BmorOBP21 | Gong et al. 2009 | MsexOBP28 | Richard et al. 2015 |
| BmorOBP22 | Gong et al. 2009 | MsexOBP29 | Richard et al. 2015 |
| BmorOBP23 | Gong et al. 2009 | MsexOBP39 | Richard et al. 2015 |
| BmorOBP24 | Gong et al. 2009 | MsexOBP30 | Richard et al. 2015 |
| BmorOBP25 | Gong et al. 2009 | MsexOBP08 | Richard et al. 2015 |
| BmorOBP26 | Gong et al. 2009 | MsexOBP31 | Richard et al. 2015 |
| BmorOBP27 | Gong et al. 2009 | MsexOBP34 | Richard et al. 2015 |
| BmorOBP29 | Gong et al. 2009 | MsexOBP35 | Richard et al. 2015 |
| BmorOBP30 | Gong et al. 2009 | MsexOBP40 | Richard et al. 2015 |
| BmorOBP31 | Gong et al. 2009 | MsexOBP36 | Richard et al. 2015 |
| BmorOBP32 | Gong et al. 2009 | MsexOBP37 | Richard et al. 2015 |

**CSPs**

| **Protein name** | **Accession number** | **Protein name** | **Accession number** |
| --- | --- | --- | --- |
| HarmCSP6 | AEX07267 | CsupCSP12 | Cao et al. 2015 |
| HarmCSP11 | Zhang et al. 2015 | CsupCSP13 | Cao et al. 2015 |
| HarmCSP13 | Zhang et al. 2015 | CsupCSP14 | Cao et al. 2015 |
| HarmCSP17 | Zhang et al. 2015 | CsupCSP15 | Cao et al. 2015 |
| HassCSP6 | Zhang et al. 2015 | CsupCSP16 | Cao et al. 2015 |
| HassCSP11 | Zhang et al. 2015 | CsupCSP17 | Cao et al. 2015 |
| HassCSP19 | Zhang et al. 2015 | CsupCSP18 | Cao et al. 2015 |
| HvirCSP1 | AAM77041 | CsupCSP19 | Cao et al. 2015 |
| HvirCSP2 | AAM77040 | CsupCSP20 | Cao et al. 2015 |
| HvirCSP3 | AAM77042 | CsupCSP21 | Cao et al. 2015 |
| HvirCSP0009 | ACX53700 | BmorCSP1 | ABH88194 |
| HvirCSP0103 | ACX53788 | BmorCSP2 | ABH88195 |
| HvirCSP0129 | ACX53813 | BmorCSP3 | ABH88196 |
| HvirCSP0056 | ACX53745 | BmorCSP4 | ABH88197 |
| HvirCSP0119 | ACX53804 | BmorCSP5 | ABH88198 |
| HvirCSP0115 | ACX53800 | BmorCSP6 | ABH88199 |
| CsupCSP1 | Cao et al. 2015 | BmorCSP7 | ABH88200 |
| CsupCSP2 | Cao et al. 2015 | BmorCSP8 | ABH88201 |
| CsupCSP3 | Cao et al. 2015 | BmorCSP9 | ABH88202 |
| CsupCSP4 | Cao et al. 2015 | BmorCSP10 | ABH88203 |
| CsupCSP5 | Cao et al. 2015 | BmorCSP11 | ABH88204 |
| CsupCSP6 | Cao et al. 2015 | BmorCSP12 | ABH88205 |
| CsupCSP7 | Cao et al. 2015 | BmorCSP13 | ABH88206 |
| CsupCSP8 | Cao et al. 2015 | BmorCSP14 | ABH88207 |
| CsupCSP9 | Cao et al. 2015 | BmorCSP15 | ABH88208 |
| CsupCSP10 | Cao et al. 2015 | BmorCSP16 | ABH88209 |
| CsupCSP11 | Cao et al. 2015 |  |  |
