# Supplementary material for: Candidate odorant binding proteins and chemosensory proteins in the larval chemosensory tissues of two closely related noctuidae moths, Helicoverpa armigera and H. assulta
Source: PLoS One. 2017 Jun 8;12(6):e0179243. doi: 10.1371/journal.pone.0179243 (PMC5464669; doi:10.1371/journal.pone.0179243)
Supplement: S2 Material — (DOCX) [file pone.0179243.s002.docx]

**S2 material. Primers for RT-PCR expression analyses of *H. armigera* OBPs and CSPs.**

| Primer | Sequences (5'to3') | Primer | Sequences (5'to3') | Product size |
| --- | --- | --- | --- | --- |
| HarmGOBP2-F | ATGACGTCGAAGAGTTGTTTGTTGC | HarmGOBP2-R | TACTTCTCCATGACGGCCTCTATCA | 485bp |
| HarmOBP1-F | TTCGTTTTGTGTGTTGTGGCTGT | HarmOBP1-R | GCTTTATGCTCCAAGAAGCATGC | 410bp |
| HarmOBP2-F­­ | CACGAGCTTGCACTCAATAATACTGT | HarmOBP2-R | CCTATTTGGTCTTCAGTCACCTGATT | 275bp |
| HarmOBP3-F | AAGTTCACTTGTTTTGTTTTGTGCG | HarmOBP3-R | TAGATGATGATCTGAGCCCTGTGC | 437bp |
| HarmOBP4-F | CCAAACTCACTTGTGTTGTTTTTGC | HarmOBP4-R | TGGAGAGCTCAGATTTATTTTCTATGAA | 435bp |
| HarmOBP5-F | ACTTGTTTAGTTTTGTGCGTTGTGG | HarmOBP5-R | ACAATGGGACCTCAGCCTTGTG | 430bp |
| HarmOBP6-F | TAAGTTCACTTGCTTGCTTCTGTGC | HarmOBP6-R | TAATCTGAGCCCTGTGTTCCAAAAA | 431bp |
| HarmOBP9-F | TGTGCAAATTCAGTGTTCTGTTCCTA | HarmOBP9-R | TCTTGCATACAACGGAATAGGACATC | 430bp |
| HarmOBP14-F | TGTCGTGTTCTGTGTTTTGGTAGCT | HarmOBP14-R | GTACCATGGTGGTAGCATTTGAAGA | 375bp |
| HarmOBP15-F | GACGAGACTTCTACTCACCATCGGT | HarmOBP15-R | CCATCGACATCAAACATCTGATGAA | 479bp |
| HarmOBP17-F | ATTCCTGCGAGCGGCAGCA | HarmOBP17-R | TATGAGGAAGTAGTCGGCCTTGTTCG | 429bp |
| HarmOBP19-F | TTTCCGTTTTTTGTTGTGTTTGTGT | HarmOBP19-R | GGCAGCTTGATACCCAACTTAGATG | 438bp |
| HarmOBP21-F | AGTTGTTAGGGCTGCTTGTAATTGC | HarmOBP21-R | TCAAGGAAAAATAAAGCTGGCTGG | 416bp |
| HarmOBP22-F | ATTTTGTTTGTAACAATTAGCTCGTCGT | HarmOBP22-R | CGGGTCGAACTCGTAAATGCAT | 384bp |
| HarmOBP23-F | TGAAGCAAGGAAGTCGGTACAGC | HarmOBP23-R | TTATGTCTTTTCGGTAGAAGCTGGTTT | 521bp |
| HarmOBP25-F | GAGATCTGTCGTGTTGTTCAACATGT | HarmOBP25-R | CGTCAGGCAAGCGTTACAGGA | 568bp |
| HarmOBP26-F | TTTAAATCATCAGTGTTTCTGTGTTGTG | HarmOBP26-R | AGCGTTCTCTGTCATGCATTTGTAA | 438bp |
| HarmOBP27-F | TGGAACTTTTTTATTGTGGTTTTAGCAT | HarmOBP27-R | TAAAGCTTAAACCCGAACTTGGGAG | 440bp |
| HarmOBP28-F | ATTTTTTTGTGTGTTCTTGTGTGGTGT | HarmOBP28-R | CACGAAGTCAAAATCAGGAGACTTTTC | 430bp |
| HarmOBP29-F | TAAGTTTTCTGGTCTCGTTTTGTGC | HarmOBP29-R | TGAGGATGCTAGTCTTGTGTTCCAA | 419bp |
| HarmOBP30-F | GAAGACGTTTGTGGTTTTGGCG | HarmOBP30-R | AATATCGCGTGTTTGGGGTCCT | 399bp |
| HarmOBP31-F | CGAAGTTCACGTGCATCGTCCT | HarmOBP31-R | GGCATCTCAGCTTTGTGTTCCAA | 442bp |
| HarmOBP33-F | TTCAAGAGCAAAGTATTTCCAGAAGG | HarmOBP33-R | AAAAGTCGATGTCAAATCCGAGCT | 298bp |
| HarmOBP35-F | TTTTGTGCTTTAATTATTGTTGCATCC | HarmOBP35-R | TTAACATTTCCATGCCGCTTCA | 423bp |
| HarmOBP36-F | GAACTCATGCTGGCAGAACAGATTC | HarmOBP36-R | CCATCTTAGCAGCAGTTTCTGTCAA | 387bp |
| HarmCSP1-F | ATGAAAGTCCTGCTAGTACTGTGCCT | HarmCSP1-R | TTATTCGGGGATCTGGATGCC | 384bp |
| HarmCSP2-F | TGAAGGTAGTGCTACTCACACTCTGCT | HarmCSP2-R | GGAAGTCTTTCCACGCCTGTTG | 351bp |
| HarmCSP4-F | ACGAGATCCTCGGCAACCGT | HarmCSP4-R | ATCAAATTGAAACTAAACGGTCGTCA | 318bp |
| HarmCSP5-F | CTTCGTAGTAGTATGTCTCCTCGGGC | HarmCSP5-R | GTGCTTAAGTTCCCTGAGTTCCTGTT | 373bp |
| HarmCSP7-F | AGTATTCGTGGTATTATCCGTCCTGA | HarmCSP7-R | AGCCACGTTCTTCAAGAGTTCGTT | 376bp |
| HarmCSP8-F | GCTATCGTGCTATGTGTGGTGGC | HarmCSP8-R | TGTTGCACTTCTTCGAGCTCCTT | 374bp |
| HarmCSP9-F | GTGGATGAAATCTTGGAGTCTCAGC | HarmCSP9-R | ATCATAAATTTCCATTTCCACCACC | 363bp |
| HarmCSP10-F | CTAACTAAACGGCTCACAAGTGCAAC | HarmCSP10-R | GTCTCTTGTGGCGTACACTGAGGAC | 320bp |
| HarmCSP12-F | GCTGGGCTTTTAAATCTTGAATCG | HarmCSP12-R | CTCTTCGTAGCCTCTAGGCAGCTC | 341bp |
| HarmCSP14-F | CGGTATTGTTTGTGTGTGCCCTT | HarmCSP14-R | CTACCGTCCGACGACCGCTC | 872bp |
| HarmCSP15-F | AAAGCCGTATTCCTACTATGCTTGG | HarmCSP15-R | TTTGAATTTGGAATTTAGGCAATTG | 386bp |
| HarmCSP16-F | AAGTTTCTTGTGGTTGCTATCGTGG | HarmCSP16-R | GTCAGCGGCAAGGAAAGACTCAT | 363bp |
| HarmCSP19-F | ATGCAATTTGAAGGTGTCAATATTGA | HarmCSP19-R | CAATAGCCTTCTCGAAGGCTTCAT | 368bp |
| HarmCSP20-F | GGAGTTGAACATGAAGACCCTGGT | HarmCSP20-R | CTAAAACGCGGCAACTGCAG | 382bp |
| HarmCSP21-F | AACTCTGCTATCGTGCTATGTGTGG | HarmCSP21-R | TAATGTTGCACTTCTTCGAGCTCCT | 389bp |
| HarmCSP22-F | AAGTCCTGATGGTAGCAGTTCTTGC | HarmCSP22-R | CTGTTCCAAGCATAAAAGCAGAGAA | 360bp |
| HarmCSP23-F | ATGAAACTCCTGATTGTTTTGGCG | HarmCSP23-R | TGGTGTTACGTTTAATGGCCGTATT | 389bp |
| HarmCSP24-F | GTACGACAACGTAGACCTGGACGA | HarmCSP24-R | TGTTGTAATGTTTGAACTACGCGTTT | 323bp |
| HarmCSP25-F | CACTTTTCGCTGAAGGGACATCTC | HarmCSP25-R | CGTGTACTTGTTGTTGGGGTCGTA | 391bp |
| HarmActin-F | AGGGTGTGATGGTCGGTATGGG | HarmActin-R | TGGCGGGGGAGTTGAAGGTT | 308bp |
