# Supplementary figures and images for: Candidate odorant binding proteins and chemosensory proteins in the larval chemosensory tissues of two closely related noctuidae moths, Helicoverpa armigera and H. assulta
Source: PLoS One. 2017 Jun 8;12(6):e0179243. doi: 10.1371/journal.pone.0179243 (PMC5464669; doi:10.1371/journal.pone.0179243)

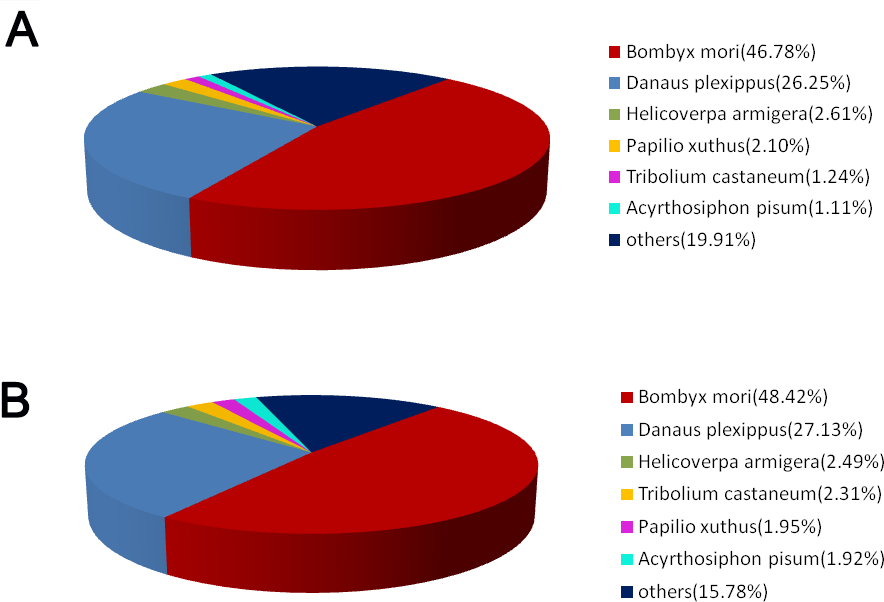

Supplement: S3 Material — (A) H. armigera unigenes. (B) H. assulta unigenes. (TIF) [file pone.0179243.s003.tif]
